# Fractional esterification rate of cholesterol in high-density lipoprotein associates with risk of coronary heart disease

**DOI:** 10.1186/s12944-017-0545-z

**Published:** 2017-08-24

**Authors:** Junmeng Liu, Ruiyue Yang, Min Zhou, Wen Mao, Hongxia Li, Haijian Zhao, Shu Wang, Wenxiang Chen, Jun Dong, Qing He

**Affiliations:** 1 Cardiology Department, Beijing Hospital, Beijing, 100730 People’s Republic of China; 2 The MOH Key Laboratory of Geriatrics, Beijing Hospital, National Center of Gerontology, Beijing, 100730 People’s Republic of China; 3 Beijing Hospital and National Center for Clinical Laboratories, Beijing, 100730 People’s Republic of China

**Keywords:** Cholesterol, Esterification rate, High density lipoprotein, Lipoprotein subfractions, Coronary heart disease

## Abstract

**Background:**

Fractional esterification rate of cholesterol in high-density lipoprotein (FER_HDL_) has been found to be closely correlated with atherosclerotic dyslipidemia, especially lipoprotein distributions, and is a potentially useful predictor for coronary heart disease (CHD). The associations of FER_HDL_, measured by the simple and precise HPLC method, with angiographically defined CHD and its related risk factors in Chinese patients were evaluated.

**Methods:**

Two hundred and fifty eight Chinese patients who had indications for angiography were enrolled in this study. Coronary angiograms were obtained by the standard techniques. FER_HDL_ was determined by the HPLC method. Cholesterol levels in serum HDL, LDL and subfractions were measured by ultracentrifugation/HPLC method. Associations between FER_HDL_ and CHD and CHD risk factors were analyzed.

**Results:**

FER_HDL_ was correlated with almost all the CHD risk factors. Compared with the non-CHD group, the CHD patients had higher values of FER_HDL_ (20.9 ± 7.9%/h vs 17.7 ± 7.1%/h, *p* = 0.001). FER_HDL_ was found to be independently and positively correlated with log TG (β = 0.386, *P* < 0.001) and log (LDLb-C) (β = 0.165, *P* = 0.020), respectively, and negatively correlated with log (HDL2-C)(β = −0.351, *P* < 0.001). Logistic regression analysis demonstrated that age, diabetes mellitus, smoking and FER_HDL_ (OR = 1.056–1.080, *p* < 0.05) were independent risk factors for CHD.

**Conclusion:**

FER_HDL_ significantly correlated with both HDL2-C and LDLb-C, and therefore, is the predictor of lipoprotein distributions. In addition, after correcting for the presence of classic risk factors, FER_HDL_ was independently associated with the presence of angiographically defined CHD.

## Background

Epidemiologic and interventional studies have clearly established an inverse association between plasma levels of high-density lipoprotein (HDL) cholesterol (HDL-C) and incidence of coronary heart disease (CHD) [1, 2]. However, contrary to what has been achieved in the field of low-density lipoprotein cholesterol (LDL-C) control through statin therapy, pharmacological modulation of HDL-C has not made a comparable success in the clinical arena. Several therapies, including nicotinic acid, fibric acid derivatives, and inhibitors of cholesteryl ester transfer protein (CETP), were not associated with CHD risk reduction despite significant increase in HDL-C levels [3]. In addition, there are several genetic syndromes of very low HDL-C that are not associated with an increased risk of premature CHD [4]. Therefore, the static measurement of HDL-C levels has inherent limitations as a metric of the functional effects of HDL and HDL associated CHD risks [5, 6]. The determination of HDL function, and not only HDL levels, may improve clinical CHD risk assessment, especially in low-risk populations.

Fractional esterification rate of HDL cholesterol (FER_HDL_) is defined as the percentage of HDL (after depleting the apoB-containing lipoproteins) free cholesterol (HDLFC) esterified during HDL incubation (ea. at 37 °C, 1 h). In the last decades, researchers have found that FER_HDL_ is significantly correlated with atherosclerotic dyslipidemia and is a marker of lipoprotein particle sizes [7, 8]. Subsequently, FER_HDL_ was shown to be a functional test of HDL quality and a strong predictor of positive findings on coronary angiography and therefore, is a potentially useful risk predictor for CHD [9–11].

FER_HDL_ has been measured by an isotopic assay established by Dobiasova [12, 13]. Although this method is reliable, the time-consuming sample preparation and the requirement of radioactive isotope make it inconvenient for use in clinical laboratories. In our previous study, we established a simple, nonradioactive, and precise HPLC method for the measurement of FER_HDL_ [14]. With this method, we have documented significant associations between FER_HDL_ and lipid profiles, especially the negative correlation with large HDL and positive correlation with small-dense LDL concentrations in healthy volunteers. Therefore, the aims of the current study are to evaluate the associations of FER_HDL_, measured by the HPLC method, with angiographically defined CHD and CHD severity, and the associations between FER_HDL_ and the major CHD risk factors in Chinese patients.

## Methods

### Study subjects

The study subjects included 258 patients hospitalized from 2008 to 2011 at Beijing Hospital, who had indications for angiography and had never received statin therapies in the preceding 6 months. Height, weight and blood pressure were measured. Baseline demographics and medical histories associated with CHD, such as smoking, hypertension (HTN), and diabetes mellitus (DM) were recorded by self-reported questionnaire. The patients who were diagnosed with acute myocardial infarction, or receiving radiotherapy or chemotherapy were excluded from this study. Patients with systolic blood pressure (SBP) of 140 mmHg or diastolic blood pressure (DBP) of 90 mmHg or higher, or receiving antihypertensive therapy were considered to have HTN. Patients recorded for DM by self-report questionnaire or with a fasting blood glucose (FBG) concentration of 7.0 mmol/L were considered to have DM. Patients with a serum total cholesterol (TC) > 6.21 mmol/L, LDL-C > 4.14 mmol/L, triglycerides (TG) >1.70 mmol/L, or HDL-C < 1.04 mmol/L, were considered to be dyslipidemia. Blood samples were collected after an overnight fast into common vacutainer tubes, serum was isolated within 2 h, aliquoted and frozen at −80 °C until analyses.

### Ethics statement

This study was reviewed and approved by the Beijing Hospital Ethics Committee. All studied individuals were informed in writing of the intended use of their samples and each provided written consent.

### Coronary angiography

Coronary angiograms were obtained by the standard techniques with multiple views recorded. Coronary arteries were divided into 15 segments, according to the classification of the American Heart Association Grading Committee. Coronary artery segments were carefully selected by two cardiologists on the basis of smooth luminal borders and the absence of stenosis. The presence of stenosis was determined using a computer-assisted coronary angiography analysis system (Mipron 1; Kontron Co., Tokyo, Japan), and CHD was defined as the existence of any one of the coronary artery or major branch stenosis ≥50% in diameter. Individuals with <50% stenosis were included into the non-CHD group. Individuals with negative findings in computed tomography of the coronary arteries or stress myocardial perfusion imaging were also included into the non-CHD group.

### Measurement of serum FER_HDL_ by HPLC

Serum FER_HDL_ was measured using our previously reported HPLC method [13]. Briefly, serum samples were thawed at room temperature and 1 mL aliquots were transferred to another set of tubes. Subsequently, 0.1 mL of precipitation reagent (10 g/L solution of dextran sulfate and 0.5 mol/L MgCl_2_) was added, vortexed for 10 s, incubated at room temperature for 15 min, and then centrifuged at 1500 g at 4 °C for 30 min. The HDL supernate was transferred and aliquoted into two tubes, with 0.4 mL per tube. One set of the tubes was placed in an ice water bath, while the other set was incubated at 37°Cwater bath for exactly 1 h and then put into ice water bath for HDLFC analyses. HDLFC was measured by our previously reported method [14, 15]. Free cholesterol and internal standard (stigmasterol) were extracted with hexane, oxidized to cholest-4-en-3,6-diones with chromic acid, and analyzed by HPLC. FER_HDL_ was presented by the percentage decrease of HDLFC mass during incubation at 37 °C for 1 h and was calculated by the following equation:

$$ {\mathrm{FER}}_{\mathrm{HDL}}\left(\%/\mathrm{hr}\right)=\left(\ {\mathrm{HDL}\mathrm{FC}}_{0{{}^{\circ}\mathrm{C}}}\hbox{--} {\mathrm{HDL}\mathrm{FC}}_{37{{}^{\circ}\mathrm{C}}}\right)/{\mathrm{HDL}\mathrm{FC}}_{0{{}^{\circ}\mathrm{C}}}\times 100\% $$

### Other parameters and laboratory assays

Cholesterol concentrations in serum HDL, LDL and subfractions (HDL-C, HDL2-C, HDL3-C, LDL-C, LDLa-C, and LDLb-C) as well as in lipoprotein (a) [Lp(a)-C] were measured by our previously established ultracentrifugation/HPLC methods [16, 17]. Serum FBG, TC, TG, apolipoprotein (apo) CII, apoCIII, high-sensitive C reactive protein (hsCRP) and uric acid (URIC) were measured enzymatically using assay kits from Sekisui Medical Technologies (Osaka, Japan) on a Hitachi 7180 chemistry analyzer.

### Statistical analyses

For continuous variables, normality was tested by Kolmogorow-Smirnov test. Variables with normal distributions were described as mean ± standard deviation (SD). The means of variables were compared by T-test between two groups and by the one-way analysis of variance among multi-groups. The variables with skewed distribution were presented as median and interquartile ranges (25th to 75th percentile). Categorical variables were presented as frequencies and percentages, and were analyzed by Chi-square test. Correlations between FER_HDL_ and other parameters were analyzed by Spearman nonparametric test. Stepwise multiple linear regression analysis was used to test the independent relationships of FER_HDL_ with the measured variables. Meanwhile, collinearity testing was used to avoid including interdependent model variables. The significance levels for entering and removing an explanatory variable were set at 0.05 and 0.10, respectively. The associations between measured variables and CHD were evaluated by multivariable logistic regression analysis. Odds ratios (ORs) for CHD versus non-CHD were estimated with the corresponding 95% confidence intervals (CIs). The odds ratios were adjusted for Age, gender, body mass index (BMI), smoking status, and the presence or absence of DM, HTN, and dyslipidemia. All reported *P* values were two-tailed, with a *P* value of 0.05 indicating statistical significance. Statistical analyses were performed with the use of SPSS software, version 17.0 (SPSS Inc.).

## Results

### Demographic and clinical characteristics of the study population

The study population consisted of 258 hospitalized patients, 170 men and 88 women, aged 30 ~ 84 years old. The Demographic and clinical characteristics were shown in Table 1. These subjects were divided into CHD and non-CHD groups according to coronary angiography results. The mean age was 64.2 y in the CHD group and 61.9 y in the non-CHD group (*P* = 0.09). There were significantly more males, higher prevalence of DM, and higher percentage of smoking in CHD cases than in the controls. Age, BMI, blood pressure, and percentage of HTN and dyslipidemia were not significantly different between the two groups.

**Table 1:** Demographic and clinical characteristics of study population

	Non CHD group (*n* = 100)	CHD group (*n* = 158)	*P*
Age, years	61.9 ± 10.6	64.2 ± 10.8	0.091
BMI, kg/m^2^	25.6 ± 3.6	25. 7 ± 3.1	0.655
SBP, mmHg	128.0 ± 16.4	130.8 ± 17.3	0.191
DBP, mmHg	76.4 ± 10.9	74.8 ± 9.9	0.248
Men, %	58 (58.0)	112 (70.9)	0.033
Smoker, %	36 (36.0)	77 (48.7)	0.045
Hypertension, %	62 (62.0)	110 (69.6)	0.206
Diabetes, %	17 (17.0)	59 (37.3)	<0.001
Dyslipidemia, %	72 (72.0)	129 (81.6)	0.069

### Univariate analyses of laboratory indexes between CHD and non-CHD groups

**Fig. 1:**
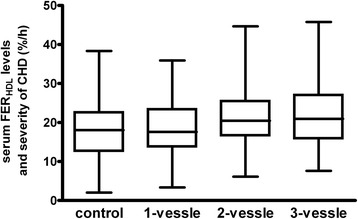
Serum FER_HDL_ levels and severity of CHD. The 158 CHD patients were divided into 1-vessle (*n* = 51), 2-vessel (*n* = 44) and 3-vessel (*n* = 63) stenosed subgroups. The box plots show the median and 25th and 75th percentiles. Whiskers in the plots represent the highest and lowest values

Results of univariate analyses of laboratory parameters between CHD and non-CHD groups were shown in Table 2. There were no significant differences for serum TC, LDL-C, LDLa-C, LDLb-C, HDL3-C, and Lp(a)-C concentrations between the two groups. Compared to the non-CHD group, the CHD group had significantly higher levels of TG and lower levels of HDL-C and HDL2-C. Serum FBG, apoCII, apoCIII, URIC and hsCRP levels were significantly higher in CHD group than in the non CHD group. FER_HDL_ was found to be significantly higher in CHD group than in the controls (20.8 ± 7.9 vs 17.7 ± 7.1%/h, *P* = 0.001). In addition, the 158 CHD patients were further divided into 1-vessle (*n* = 51), 2-vessel (*n* = 44) and 3-vessel (*n* = 63) stenosed subgroups and the above parameters were compared among groups. As shown in Fig. 1, with the increase of the number of stenosed vessels, FER_HDL_ was significantly increased (*P* = 0.004).

**Table 2:** Univariate analyses of laboratory indexes between two groups

	Non CHD group (*n* = 100)	CHD group (*n* = 158)	*P*
TC, mmol/L	4.55 ± 0.87	4.60 ± 0.98	0.662
TG ^a^, mmol/L	1.30 (1.00 ~ 1.62)	1.60 (1.20 ~ 2.20)	<0.001
LDL-C ^a^, mmol/L	2.45 (2.12 ~ 2.96)	2.33 (1.94 ~ 2.95)	0.483
LDLa-C, mmol/L	2.17 ± 0.65	2.11 ± 0.73	0.285
LDLb-C ^a^, mmol/L	0.26 (0.19 ~ 0.38)	0.28 (0.19 ~ 0.46)	0.350
HDL-C ^a^, mmol/L	1.03 (0.88 ~ 1.25)	0.96 (0.82 ~ 1.18)	0.017
HDL2-C ^a^, mmol/L	0.55 (0.43 ~ 0.72)	0.50 (0.40 ~ 0.65)	0.022
HDL3-C ^a^, mmol/L	0.48 (0.43 ~ 0.53)	0.46 (0.40 ~ 0.52)	0.063
Lp(a)-C ^a^, mmol/L	0.06 (0.03 ~ 0.11)	0.06 (0.04 ~ 0.11)	0.559
Apo CII ^a^, mg/dL	3.80 (2.40 ~ 4.80)	4.10 (2.80 ~ 5.45)	0.046
Apo CIII ^a^, mg/dL	7.50 (6.20 ~ 9.10)	8.50 (6.60 ~ 10.50)	0.033
hsCRP ^a^, mg/ dL	0.12 (0.05 ~ 0.24)	0.18 (0.08 ~ 0.28)	0.033
FBG ^a^, mmol/L	5.00 (4.70 ~ 5.60)	5.30 (4.88 ~ 6.10)	0.028
URIC ^a^, umol/L	329.0 (260.0 ~ 378.0)	355.0 (306.5 ~ 412.5)	0.002
FER_HDL_, %/h	17.7 ± 7.1	20.8 ± 7.9	0.001

For apoCII and apoCIII, 125 cases and 75 controls were analyzed

^a^ Median (Q1 ~ Q3)

### Correlations between FER_HDL_ and other parameters

Univariate correlations between FER_HDL_ and clinical and laboratory parameters were analyzed and the results were shown in Table 3. FER_HDL_ values were significantly and positively correlated with BMI (*P* < 0.001), serum FBG (*P* < 0.001), TG (*P* < 0.001), LDLb-C (*P* < 0.001), apoCII (*P* < 0.001), apoCIII (*P* < 0.001), Lp(a) (*P* = 0.005), hsCRP (*P* = 0.001), and URIC (*P* < 0.001), respectively. On the other hand, significantly negative correlations between FER_HDL_ and age (*P* < 0.001), HDL-C (*P* < 0.001), HDL2-C (*P* < 0.001) HDL3-C (*P* < 0.001) and LDLa-C (*P* = 0.005) were observed respectively.

**Table 3:** Univariate correlations of FER_HDL_ with clinical and laboratory parameters

	FER_HDL_			FER_HDL_	
	r	P		r	P
Age	−0.302	<0.001	TC	−0.009	0.891
BMI	0.319	<0.001	TG	0.619	<0.001
SBP	0.086	0.169	LDL-C	−0.052	0.406
DBP	0.066	0.294	LDLa-C	−0.174	0.005
FBG	0.281	<0.001	LDLb-C	0.429	<0.001
URIC	0.275	<0.001	HDL-C	−0.643	<0.001
Apo CII	0.447	<0.001	HDL2-C	−0.699	<0.001
Apo CIII	0.352	<0.001	HDL3-C	−0.279	<0.001
hsCRP	0.213	0.001	Lp(a)-C	0.179	0.005

### Multiple linear regression model

For multivariate reevaluation of the univariate correlations, all variables given in Table 3 were entered into a stepwise multiple linear regression analysis as independent variables to identify significant contributors to the distribution of FER_HDL_. The relationships between FER_HDL_ and other CHD risk factors were shown in Table 4. FER_HDL_ was found to be independently and positively correlated with logTG (β = 0.386, *P* < 0.001), and log(LDLb-C) (β = 0.165, *P* = 0.020), respectively, and negatively correlated with age (β = −0.152, *P* = 0.017) and log(HDL2-C)(β = −0.351, *P* < 0.001).

**Table 4:** Multiple linear regression analysis with FER_HDL_ as the dependent variable

Variables	Regression coefficient	SE	β	*P*
Age	−0.093	0.038	−0.152	0.017
Log (HDL2-C)	−19.259	3.653	−0.351	<0.001
Log (LDLb-C)	4.326	1.832	0.165	0.020
Log TG	16.037	2.746	0.386	<0.001

β indicates the standardized partial regression coefficient. Adjusted R^2^ = 0.453, *P* = 0.017

All variables given in Table 3 were entered into a stepwise multiple linear regression analysis as independent variables. Collinearity testing was used to avoid including interdependent model variables. *P* values for entry and removal, 0.05 and 0.10, respectively

### Logistic regression model

Logistic regression analysis was carried out to further quantify the association between FER_HDL_ and CHD, and the results were shown in Table 5. Multivariate adjustment was used to control for traditional CHD risk factors, including age, gender, BMI, smoking, DM, hypertension, and dyslipidemia. After this multivariate adjustment, each 1 SD increase in FER_HDL_ value was associated with an approximately two-fold increase in the risk of CHD (OR = 1.716, 95% CI: 1.254–2.348, *P* < 0.001). As compared with subjects in the lowest quartile of FER_HDL_ levels, the OR (95% CIs) for CHD risk in subjects belonging to quartiles 2, 3, and 4 were 2.447 (1.150–5.204), 1.553 (0.734–3.282), and 3.174 (1.408–7.155), respectively (*P* trend = 0.022). Age, smoking and DM were also independently correlated with the risk of CHD, and FER_HDL_ was the only lipid related risk factor for CHD.

**Table 5:** Logistic regression model for the case-control study

	Univariable		Multivariable ^a^	
	OR (95% CI)	*P*	OR (95% CI)	*P*
Age	1.242 (0.965 ~ 1.599)	0.092	1.646 (1.203 ~ 2.252)	0.002
Smoke	1.298 (1.005 ~ 1.676)	0.045	1.451 (1.083 ~ 1.943)	0.013
DM	1.638 (1.232 ~ 2.177)	0.001	1.576 (1.177 ~ 2.110)	0.002
FER_HDL_ as a continuous variable				
Per SD increment	1.556 (1.181 ~ 2.050)	0.002	1.716 (1.254 ~ 2.348)	0.001
FER_HDL_ as a categorical variable				
First quartile	1.0 (referent)	---	1.0 (referent)	---
Second quartile	2.230 (1.092 ~ 4.557)	0.028	2.447 (1.150 ~ 5.204)	0.020
Third quartile	1.285 (0.641 ~ 2.573)	0.480	1.553 (0.734 ~ 3.282)	0.249
Fourth quartile	2.943 (1.404 ~ 6.170)	0.004	3.174 (1.408 ~ 7.155)	0.005
*P*-value for trend	0.015		0.022	

^a^ Set of independent variables: Age, gender, BMI, smoking, diabetes mellitus, hypertension, dyslipidemia and FER_HDL_ levels

## Discussion

FER_HDL_ measures the rate of HDL cholesterol esterification which is catalyzed by the Lecithin:cholesterolacyl transferase (LCAT) in vitro. LCAT is a plasma enzyme that esterifies free cholesterol, primarily at the surface of the HDL particle, after which the cholesteryl ester molecules migrate into the inner core of the lipoprotein for metabolism [18]. As an important enzyme involved in the reverse cholesterol transport (RCT), LCAT plays a key role in the maturation of HDL particles and the RCT process [19]. Studies have suggested that the preferred substrates for LCAT are Preβ- and small sized- HDL, and the smaller the HDL particles, the faster their surface free cholesterol esterified, and FER_HDL_ reflects HDL particle distributions [10, 11]. The relationship between FER_HDL_ and LDL particle size is currently unknown. However, the metabolism of HDL and LDL are closely connected, and TG might be an important mediator between HDL and LDL. Therefore, FER_HDL_ not only reflects LCAT activity in specific conditions, but also reflects the maturation of HDL, the balance between uptake of cholesterol and transport of cholesterol esters, as well as the efficiency of RCT. Unlike the static HDL-C levels, FER_HDL_ might serve as a functional test of lipoprotein quality and HDL metabolism. Although the associations of FER_HDL_ with lipoprotein distribution and CHD risks have been quite consistent in previous studies, clinical application of FER_HDL_ was hindered by the tedious and radioactive assay method. In our previous study, we reported a simple, precise, and nonradioactive method for the measurement of FER_HDL_, and confirmed the relationship between FER_HDL_ and lipid profiles in apparently healthy subjects [14]. In the present study, we investigated the associations of serum FER_HDL_ with angiographically defined CHD, CHD severity and CHD risk factors in Chinese patients. As statin therapy significantly affects lipid profiles, the use of statins was strictly excluded in our study.

We first analyzed the correlations of FER_HDL_ with classic CHD risk factors and several other variables, and found that FER_HDL_ was positively correlated with male gender, smoking, BMI, serum FBG, TG, and negatively correlated with HDL-C, which was in accordance with previous findings [13, 14]. HDL and LDL are highly heterogeneous, and the concept that certain subfractions may be better predictors of CHD risk is of great concern. Many population studies have suggested that large HDL2 may be more cardioprotective than smaller sized HDL3 [20, 21]. However, there are also inconsistencies, with reports suggesting that HDL2 and HDL3 are equally cardioprotective [22]. In our study, FER_HDL_ showed significantly negative correlation with both HDL2-C and HDL3-C, in which correlation with HDL2-C remained significant in multiple linear regression models. Prospective studies have reported the small LDL phenotype to be an important predictor for subsequent cardiovascular diseases [23, 24]. In accordance with previous findings, a significantly positive correlation between FER_HDL_ and LDLb-C, but not LDLa-C, was observed. These results confirmed that FER_HDL_ is a reflection of lipoprotein subfraction distributions. Furthermore, FER_HDL_ was also positively associated with BMI, FBG, Uric acid, apoCII, apoCII, and hsCRP. These results suggested that FER_HDL_ is associated with almost all known CHD risk factors, and is a potentially valuable risk factor for predicting atherosclerosis and CHD.

In contrary with some of the previous studies, negative correlation between serum FER_HDL_ and age, as well as TG and age (*r* = −0.157, *p* < 0.05), was observed. In this study, 90% of the subjects were above 50y and there were more males than females. Our previous investigation showed that TG levels in Chinese males, but not in females, decreased after age of 50y [25], which might be associated with decreased FER_HDL_ values in males. The negative correlation found between FER_HDL_ and age may have resulted, by some extent, from the averagely old age and gender differences. Significantly positive correlation between FER_HDL_ and age was observed in our previous study with healthy volunteers from age 19–72 [14].

We have shown that individuals with positive angiographic findings tended to be older, smoking, with higher prevalence of DM and higher FER_HDL_ levels. In this study, logistic regression analysis showed that FER_HDL_, age, DM and smoking were independent risk factors for angiographically proven CHD, and FER_HDL_ was the only lipid related risk factor for CHD. These findings were consistent with those found by previous reports although our study sample is apparently different in ethnicity, BMI, and probably other culture-related aspects, such as lifestyle and diet. In addition, we also found that FER_HDL_ was associated with the severity of CHD.

The potential mechanisms involved in the relationship of FER_HDL_ and lipid profiles are not clear. If high FER_HDL_ does indeed reflect a high endogenous esterification rate in the HDL fraction, this finding disagrees with the generally accepted opinion that LCAT activity is beneficial to the process of RCT. Another possible explanation is that high FER_HDL_ may reflect low endogenous LCAT activity. The potential mechanism is that, due to some adverse effect (e.g. lipoproteins), endogenous LCAT activity is low, and as a result, small HDL particles accumulate. When leaving these unfavorable factors (in vitro HDL fraction), LCAT activity increases due to sufficient amount of small HDL particles. This hypothesis requires further investigation.

The following limitations of our study should be considered. First, the control subjects were defined as patients with <50% narrowing of their coronary arteries. These control subjects generally had higher CHD risks and were not truly healthy individuals, which may have underestimated the association between FER_HDL_ and CHD and limited the power of this study. Second, although our results remained consistent after multiple adjustments, we cannot exclude the possibility of residual confounding because some information was not recorded, including family history of CHD, lifestyle, stress, and other possible risk factors for CHD. Third, since this study was a case-control design, the findings need to be confirmed in future prospective studies.

## Conclusions

In conclusion, by using a simple and nonradioactive HPLC method, we demonstrated that FER_HDL_ significantly correlated with both HDL2-C and LDLb-C, and therefore, is a predictor of lipoprotein distributions. After correcting for the presence of classic risk factors and several other variables, FER_HDL_ was found to be independently associated with the presence of angiographically proven CHD.

## Abbreviations

CHD: Coronary heart disease
DM: Diabetes mellitus
FBG: Fasting blood glucose
FER_HDL_: Fractional esterification rate of cholesterol in high density lipoprotein
HDL2: High-density lipoprotein of d 1.063–1.125 kg/L
HDL3: High-density lipoprotein of d 1.125–1.210 kg/L
HDL-C: HDL-cholesterol
HDLFC: HDL free cholesterol
HTN: Hypertension
LDLa: Large-buoyant LDL
LDLb: Small-dense LDL
LDL-C: LDL-cholesterol
URIC: Uric acid
